# Synergistic Strength–Ductility Improvement in an Additively Manufactured Body-Centered Cubic HfNbTaTiZr High-Entropy Alloy via Deep Cryogenic Treatment

**DOI:** 10.3390/mi15080937

**Published:** 2024-07-23

**Authors:** Zhuoheng Liang, Zhanggen Ye, Chunfeng Liu, Liangbo Sun, Yongzhong Zhang

**Affiliations:** 1 GRINM Group Corporation Limited, National Engineering & Technology Research Center for Non-Ferrous Metals Composites, Beijing 101407, China; 2 GRINM Metal Composites Technology Co., Ltd., Beijing 101407, China; 3 General Research Institute for Nonferrous Metals, Beijing 100088, China; 4 Center of Analysis, Measurement and Computing, Harbin Institute of Technology, Harbin 150001, China; 5 School of Materials Science and Engineering, Harbin Institute of Technology, Harbin 150001, China

**Keywords:** laser melting deposition, high entropy alloy, deep cryogenic treatment, 3D printing, defects, strength–ductility synergistic improvement

## Abstract

HfNbTaTiZr high-entropy alloy has wide application prospects as a biomedical material, and the use of laser additive manufacturing can solve the forming problems faced by the alloy. In view of the characteristics of the one-time forming of additive manufacturing methods, it is necessary to develop non-mechanical processing modification methods. In this paper, deep cryogenic treatment (DCT) is first applied to the modification of a HEA with BCC structure, then the post-processing method of DCT is combined with laser melting deposition (LMD) technology to successfully realize the coordinated improvement of forming and strength–ductility synergistic improvement in lightweight Hf_0.25_NbTa_0.25_TiZr alloy. The final tensile strength of the alloy after DCT treatment is 25% higher than that of the as-cast alloy and 11% higher than that of the as-deposited alloy, and the elongation is increased by 48% and 10%, respectively. In addition, DCT also achieves induced phase transition without additional deformation.

## 1. Introduction

High-entropy alloys (HEAs) are special types of alloys with five or more principal elements and an atomic ratio of 5 to 35 at.%, which have received extensive attention in recent years [1,2,3]. Among them, Hf-Nb-Ta-Ti-Zr HEAs have attracted more and more attention as potential high-temperature structural materials and biomedical materials due to their high melting point caused by the properties of their main elements, their excellent chemical stability, and their corrosion resistance in various external environments [4,5]. Most interestingly, as single-phase body-centered cubic (BCC) solid solution alloys, Hf-Nb-Ta-Ti-Zr alloys have strong ductility. The plasticity of this BCC alloy system comes from the Jahn–Teller effect, and the existence of this effect makes the body-centered cubic unit cell deform in a specific direction, resulting in symmetry breaking, and then more slip systems are obtained [6]. Due to this characteristic, the alloy has different characteristics from the general BCC structure alloy, including the form of screw dislocation motion, and presents some face-centered cubic structure characteristics [7,8]. On the other hand, the latest research found that the plasticity of HfNbTaTiZr high-entropy alloy can be improved by increasing the lattice distortion of the alloy [9]. The above characteristics mean more modification methods can be applied to Hf-Nb-Ta-Ti-Zr alloys.

At present, the casting forming of HEAs is facing some problems due to their high melt viscosity, and laser additive manufacturing (AM) technology can effectively solve this problem [10,11,12]. Laser melting deposition (LMD) technology has high energy density and complex-shape-forming ability, which can solve the problem of HEA forming and accumulate thermal stress in as-deposited alloy samples [13]. At the same time, deep cryogenic treatment (DCT) has been gradually applied as a method to uniformly aggravate the residual stress generated by LMD, and remarkable results have been achieved [14].

In previous studies, the DCT method has often been used to modify FCC structural metals by constructing twins [15,16]. In this paper, LMD forming and DCT treatment were carried out on the previously designed lower-density Hf_0.25_NbTa_0.25_TiZr alloy in which the atoms of Hf and Ta are 25% of the atoms of other elements, in order to achieve a strength–ductility synergistic improvement by increasing the internal stress and lattice distortion so as to make up for the performance degradation caused by the decrease in density compared with the equiatomic ratio system. In this work, the DCT method was applied for the first time to the modification of BCC HEAs.

## 2. Experimental Section

**Figure 1:**
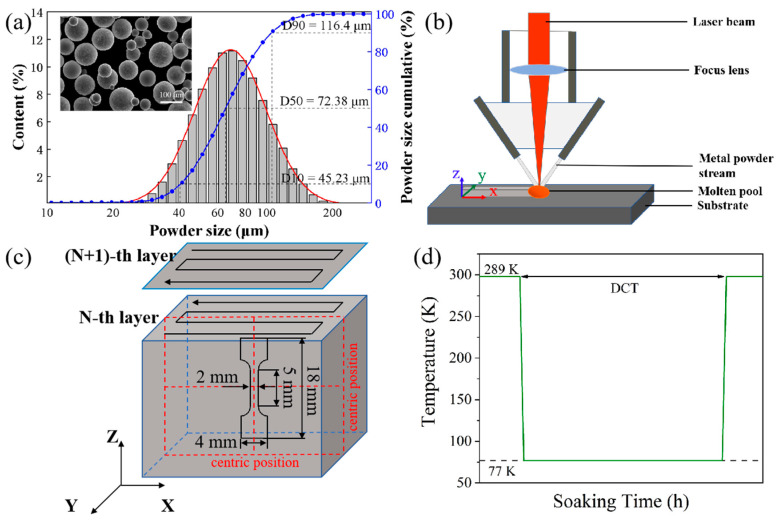
Raw materials, sample preparation methods, and DCT process schematics: (**a**) morphology and particle size of powder, (**b**) diagram of LMD system, (**c**) diagram of LMD forming and sampling, (**d**) cryogenic treatment temperature curve.

The Hf_0.25_NbTa_0.25_TiZr alloy powder used in this study was prepared by the plasma rotation electrode process (PREP). The prepared HEA powder for laser additive manufacturing with a particle size of 30–150 μm (D10 = 45.23 μm, D50 = 72.38 μm, D90 = 116.4 μm) had good sphericity. The morphology and particle size statistics of the powder are shown in Figure 1a. The processing parameters for laser melting deposition included: a laser power of 1300 W, scanning rate of 4.5 mm/s, powder feeding rate of 8 g/min, and single lift of 0.3 mm (Figure 1b). Based on this, Hf_0.25_NbTa_0.25_TiZr alloy samples with a size of 30 × 30 × 20 mm^3^ (x × y × z) were obtained, as shown in Figure 1c.

The as-deposited samples were cryogenically treated in liquid nitrogen. The temperature curve is shown in Figure 1d, and the soaking time was 12 h, 24 h, 48 h, and 120 h. In the experiment, Keller reagent was used to etch the sample along the z direction for 10 mm, and then the xoy sections without stress effects at the center height of the as-deposited and DCT samples were obtained. The central area of the sections was identified using X-ray diffraction (XRD, SmartLab 9 kW, Rigaku, Japan). The microstructure and tissue profiles of the HEA samples were observed and investigated using a field-emission scanning electron microscope (SEM, JSM-7900F) and transmission electron microscope (TEM, FEI Tecnai F20). The sampling position and the observation area were the yoz cross-section at the center of the samples. The tensile mechanical performance test was carried out with an NT100 electronic tensile machine at a constant displacement speed of 0.3 mm/min. The sampling test was carried out in the middle of the sample. The size and sampling positions of the tensile mechanical properties test sample are shown in Figure 1c, and the thickness was 1 mm. As shown in Figure 1c, the tensile specimen was taken from the center of the central section of the alloy to make the final test results reliable.

The stress change in the DCT process was analyzed by finite element (FE) simulation. A nonlinear FE model was established using commercial software ABAQUS (Abaqus/CAE 2021) for the coupled thermo-mechanical simulation of the LMD process, and the stress development during the DCT process was simulated using a visco-elastic-plastic model. A Gaussian-distributed moving heat flux was employed via the ABAQUS subroutine DFLUX. The moving direction and velocity of the heat flux were controlled by a subroutine, and elements were activated sequentially using a “birth and death” technique as the heat flux moved. The DC3D8 and C3D8T elements were used for the heat transfer analysis and stress analysis of the LMD process, respectively.

## 3. Results and Discussion

### 3.1. The Effect of Cryogenic Treatment on Residual Stress of the Alloy

**Figure 2:**
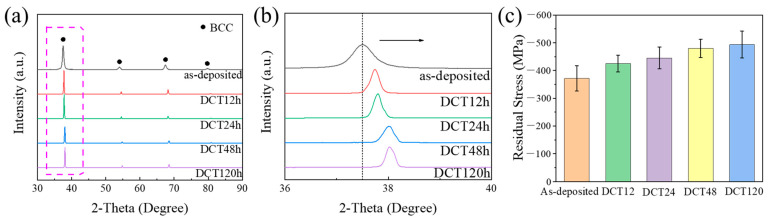
XRD test results and residual stress calculation results: (**a**) XRD diffraction patterns of samples with different soaking times, (**b**) main diffraction peak amplification diagram, (**c**) calculated value of residual stress.

According to the diffraction peak obtained by the XRD test (Figure 2a), all of the spectra display only a typical BCC structure. The enlarged image of the main peak shows that as the soaking time prolongs (Figure 2b), the position of the main peak shifts to the right, indicating that the lattice distortion of the alloy increases, and the residual stress increases. The residual stress values were calculated via the sin^2^ψ method as follows [17]:

(1) $$\sigma_{s}=\frac{E}{\left(1+v\right)}\frac{\partial (d_{\Psi }/d_{0})}{\partial ({sin}^{2}\Psi )}$$

where the Young’s modulus E of the alloy is 210 GPa [18], υ is Poisson’s ratio, ψ is the off-axis angle, $d_{\Psi }$ is the lattice spacing in the stressed state (as-deposited and DCT samples), and $d_{0}$ is that in the stress-free (an annealed as-cast sample) state. The average value of each sample was calculated five times. The calculation results show that DCT has an effect on enhancing the residual stress of the alloy (Figure 2c). It can be intuitively found from the graphic results that with an increase in soaking time, the compressive residual stress shows a gradual upward trend, and the compressive residual stress of the DCT120 sample is the highest among all samples, about −493.25 MPa. Combined with the XRD test results, the results shown in Figure 1 show that the DCT process has an effect on the lattice arrangement (interplanar spacing) of the alloy and effectively improves the residual stress level inside the alloy.

### 3.2. The Effect of Stress Aggregation on the Microstructure and Properties of the Alloy

**Figure 3:**
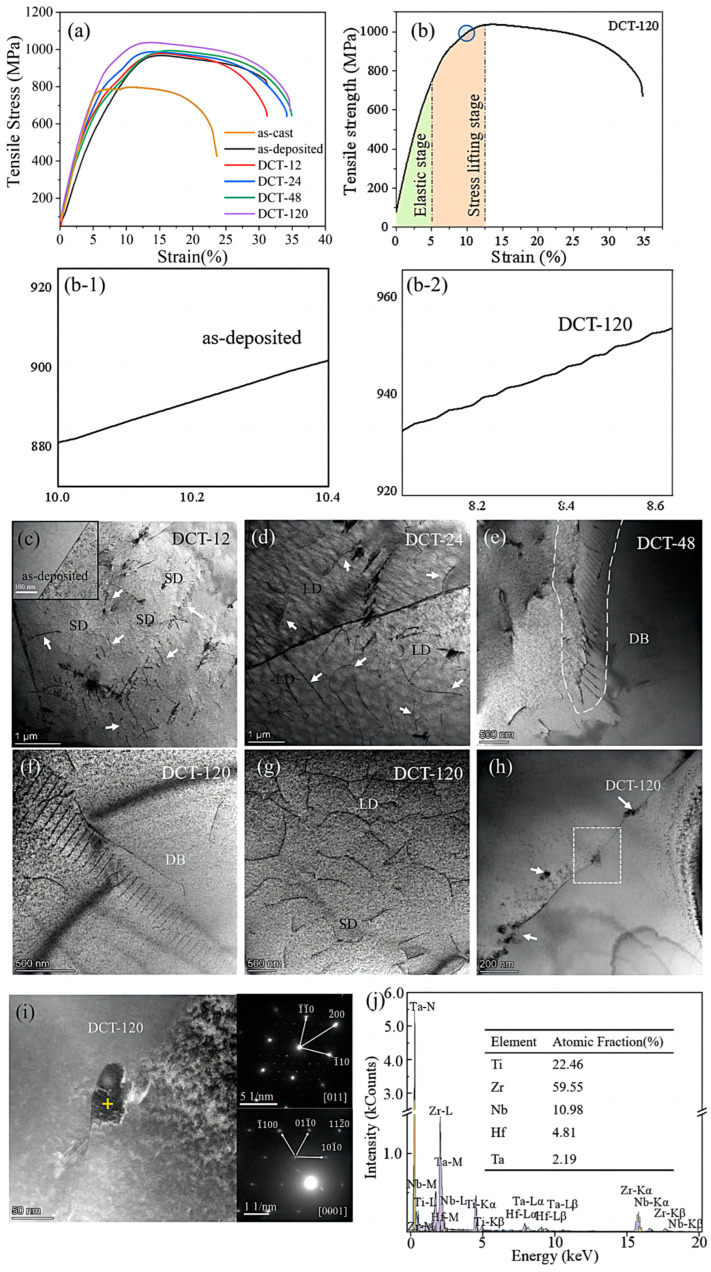
Mechanical properties and microstructure of samples at different soaking times at room temperature: (**a**,**b**) tensile mechanical properties, (**c**–**g**) the microstructure of alloys with different DCT durations, (**h**) the phase precipitation of DCT120 sample, (**i**) amplification diagram and diffraction spots of precipitated phase, (**j**) element composition of precipitated phase.

According to the test results shown in Figure 3a, LMD and DCT can simultaneously improve the strength and plasticity of the alloy. The strength improvement of DCT120 is more significant as tensile strength increased from 812 MPa to 1065 MPa and elongation increased from 24% to 36% compared to those of the as-cast sample. The yield strength of the sample after DCT is slightly higher than that in the as-deposited state, but it has a higher elongation. The increase in elongation shows the positive effect of a lattice arrangement change on the plasticity of the alloy, shown in Figure 2b, while the change in yield strength shows the particularity and complexity of the principle of plasticity improvement. Recent studies found that for Hf-Nb-Ta-Ti-Zr alloys, the increase in lattice distortion reduces the difference in the movement rate of edge dislocations and screw dislocations in the alloy, thereby improving the plasticity of the alloy [9]. A decrease in the lattice constant of single-phase multi-principal-element solid solution is inevitably accompanied by tan increase in lattice distortion, which is affected by the difference in atomic radius. In general, this increases the yield strength of the alloy. At the same time, the lattice distortion on the movement rate of different types of dislocations after the yield improves the elongation of the alloy. They were considered together to form the theoretical basis for the change in alloy properties shown in Figure 3a. The significant increase in elastic modulus indicates that DCT can densify the as-deposited alloys. The plastic stage of the alloy sample after yielding exhibits a stress-increase stage, and a serration behavior curve can be observed, which is different from the smooth curve of the deposited alloy.

According to the TEM images, compared with the as-deposited alloy, the structure of DCT-treated alloy shows observable short dislocations (SDs) (Figure 3c). With the extension of soaking time, the length of the dislocation line first increases to long dislocation (LD) (Figure 3d), and gradually develops into a dislocation band (DB) (Figure 3e). When the soaking time reached 120 h, both extensive DB distribution (Figure 3f) and dense SD (Figure 3) were observed in the alloy.

The occurrence of dislocations confirms the previously calculated values, which proves that DCT does increase the residual stress and then affects the microstructure of the Hf_0.25_NbTa_0.25_TiZr alloy. The development of dislocation distribution is also consistent with the trend in the mechanical properties shown in Figure 3a. The appearance of dislocation explains the cause of the serrated shape shown in Figure 3b, which is dislocation interaction. On the other hand, the interaction between the newly generated dislocations in the plastic deformation stage and the dislocations generated by DCT form the stress-increase stage in the alloy, as shown in Figure 3b after the yield.

Phase precipitation behavior at the grain boundary was observed in the alloy structure after 120 h of DCT (Figure 3h). As shown in Figure 3i, the precipitated phase is an HCP phase, while the matrix is a BCC single-phase solid solution. The energy spectrum analysis confirms that this phase is a Ti and Zr-rich phase and contains a certain amount of Hf (Figure 3j). The stress-driven phase transformation behavior has been reported in HfNbTaTiZr alloys but also depends on large deformation [19]. The accumulated residual stress of DCT can replace the large deformation and promote stress-induced phase transition. The formation of the precipitated phase results in a larger increase in the strength of DCT120.

Based on the results shown in Figure 2c, DCT can introduce high compressive residual stress in a sample, which has a positive effect on the tensile properties of the alloy. On the other hand, we believe that the residual stress value is the embodiment of the internal stress and microstructure changes of in alloy during DCT. The DCT process causes complex stress changes in the alloy, which result in the defect growth and phase precipitation shown in Figure 3c–i, and they provide strength enhancement. At the same time, the residual stress corresponds to the change in the interplanar spacing, while for the Hf-Nb-Ta-Ti-Zr single-phase BCC solid solution alloy, a decrease in the interplanar spacing also means an increase in the lattice distortion degree, which brings about the improvement in the plasticity of the alloy.

**Figure 4:**
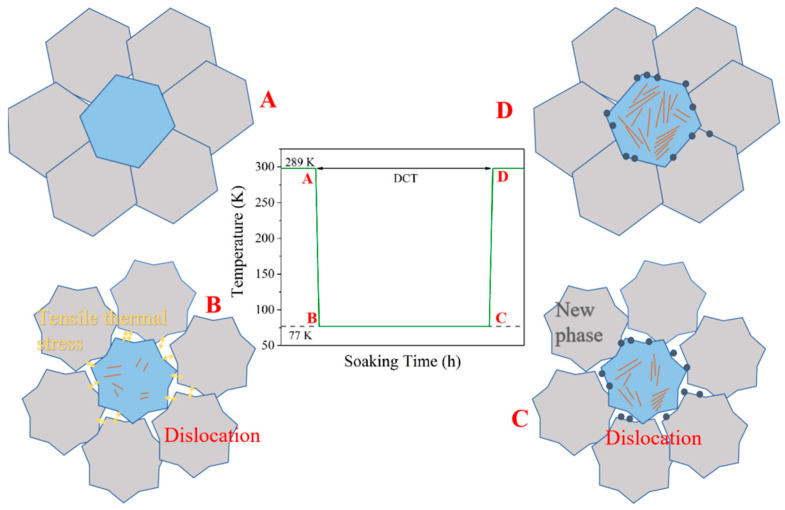
Schematic diagram of defect and residual stress formation during the DCT process: (**A**) Before DCT, (**B**) The beginning of DCT process, (**C**) The DCT process continues, (**D**) DCT process ends back to room temperature.

In view of the influence of residual stress on grain structure, it is considered that a sharp decrease in alloy temperature causes the grain to shrink, which constructs the tensile stress state between grains, assisted by the mutual restraint of the grains (Figure 4A,B). In the process from B to C, the line defects inside the grains are generated under the action of continuous tensile stress and expand with the increase in soaking time. The accompanying plastic deformation partially relaxes the tensile thermal stress. When the soaking time increases to a certain extent, the tensile stress between the crystals promotes stress-induced phase transition. Then, as the HEA sample is reheated back to room temperature at the end of the DCT process (states C to D), due to the crystalline defects and plastic deformation, as well as the intergranular constraints, a state of compressive residual stress remains in the particles.

In order to provide a further basis for this hypothesis, the finite element simulation (FE simulation) method based on ABAQUS software was used to simulate the internal stress of the alloy during LMD and DCT processes.

**Figure 5:**
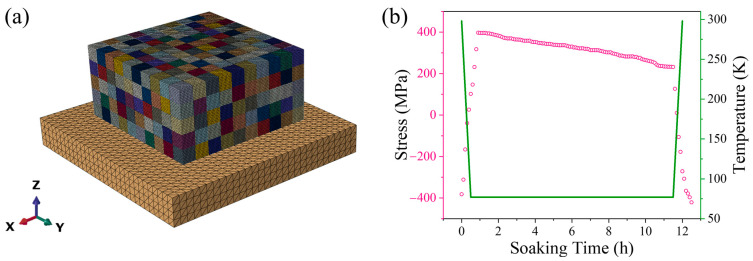
(**a**) The model and (**b**) simulation results used in the numerical ABAQUS simulation of residual stress in DCT process.

Firstly, an FE model with a dimension of 30 × 30 × 20 mm was established in which the finite element mesh used for the 5-layer and 50-track bulk HEA component was established and is shown in Figure 5a. The “element birth and death” technique was used to restore the layer-by-layer deposition behavior of the alloy during the LMD process when DC3D8 and C3D8T elements were separately employed for heat transfer analysis and stress analysis in this continuous 3D model. The substrate material parameters in the simulation process were set according to the TC4 alloy as in the actual forming process, and the material properties of the deposited material were set with the change in temperature [20]. The moving heat flux of the Gaussian distribution was set up using the ABAQUS subroutine DFLUX to simulate the process of alloy deposition with laser moving in the LMD process.

The influence range of the moving heat flux and the heat conduction in this range were defined to make the FE simulation process closer to the actual situation. The heat conduction equation in the process of additive manufacturing is [21]

(2) $$\rho (T)c_{P}(T)\frac{\partial T}{\partial t}=\nabla \cdot (k(T)\nabla T)+q_{L}$$

where ρ = 7.83 g/cm^3^ is the alloy density, T is the temperature, c_p_(T) and k(T) are the variations in specific heat capacity and thermal conductivity with temperature [20], and q_L_ is the heat flux density of the external heat source. Therefore, it is necessary to calculate and set q_L_ [22]:

(3) $$q_{L}(x,y,z)=\frac{2AP}{\pi r^{2}\eta }exp[-2\frac{{\left(x-x_{0}\right)}^{2}+{\left(y-y_{0}\right)}^{2}}{r^{2}}]exp(-\frac{\left|z-z_{0}\right|}{\eta })$$

where A = 0.83 is the laser absorption coefficient of alloy powder, *p* = 1200 W is the heat source (laser) power, r = 1.5 mm is the radius of laser beam, and η = 1.15 mm is the laser penetration depth. The boundary conditions for heat transfer are described as [23] follows:

(4) $$q_{cond}=q\left(x,y,z\right)-q_{c}-q_{r}$$

where heat is lost through conduction q_cond_, convection q_c_, and radiation q_r_ during the LMD process.

By adjusting the analysis step to match the “element birth and death” with the moving heat flux, the LMD forming process was realized using FE simulation. The intergranular residual stresses at the center of the as-deposited model obtained by FE simulation were taken as the initial stress values for the subsequent DCT process simulation. The intergranular residual stress value was 301.45 MPa compressive stress, and the stress type was consistent with the general situation obtained by the current LMD process [24,25].

Based on the existing simulation, the change in intergranular residual stress in the DCT process was simulated. The constitutive equations of elastic, plastic, thermal, and viscous strains of a visco-elastic-plastic model with initial residual stress were solved to simulate the expansion and contraction of the alloy during DCT at different temperatures. The increase in total strain with time is expressed as [26]

(5) $$\varepsilon_{total}=\varepsilon_{p}+\varepsilon_{e}+\varepsilon_{T}+\varepsilon_{V}$$

where ε_total_, ε_p_, ε_e_, ε_T,_ and ε_V_ are the total strain, plastic strain, elastic strain, thermal strain, and viscous strain, respectively. The constitutive equation can be written as [27] follows:

(6) $${d\varepsilon }_{kl}^{p}=d\lambda \frac{\partial f}{{\partial \sigma }_{ij}}$$

(7) $$\varepsilon_{kl}^{e}=\sigma_{ij}^{e}\cdot E{\left(T\right)}^{-1}$$

(8) $$\varepsilon_{kl}^{T}=\alpha_{ij}(T-T_{\infty })$$

(9) $$\varepsilon_{kl}^{V}=Aq^{n}t^{m}$$

where f is the flow area capability; λ is a constant that depends on the properties of the material; α_ij_ is the coefficient of thermal expansion; T_∞_ is the reference temperature; q is the equivalent uniaxial deviatoric stress; A, n, and m are the material constants; and t is the soaking time. Finally, the equivalent thermal shrinkage is introduced to redistribute the strain and stress inside the model, described as [28]

(10) $$\varepsilon_{T}=\alpha \cdot ∆T$$

where ε_T_ is the equivalent thermal strain, α is the thermal expansion coefficient, and ∆T is the temperature gradient.

The intergranular residual stress at the center of the FE model of the alloy at different stages was studied to obtain the results shown in Figure 5b. It can be seen from the graphic results that at the beginning of the DCT process, the stress value in the alloy changes from negative to positive rapidly due to rapid temperature reduction. This phenomenon indicates that the intergranular stress changes from compressive stress to tensile stress, which corresponds to the shrinkage of alloy grains at low temperature. As the soaking continues, the tensile stress value decreases to a certain extent; that is, a slow stress release occurs. The generation of this process is determined by the visco-elastic-plastic properties of the model and corresponds to the defect density increase and phase precipitation behavior shown in Figure 3. When the DCT process ends, the temperature of the model increases rapidly, which corresponds to the actual water quenching process, and the intergranular residual stress is transformed into compressive stress again, which has a value higher than in the initial state. Higher residual stress, on the one hand, is considered to be able to improve plasticity by inhibiting the formation of crack sources and the propagation of cracks [29]. On the other hand, it aggravates the distortion of grains in Hf_0.25_NbTa_0.25_TiZr alloy, which improves the plasticity of the alloy [9]. The simulation result here confirms the mechanism proposed in Figure 4.

## 4. Conclusions

In summary, the DCT method effectively increases the participating stress value accumulated in the LMD process, further induces the lattice distortion of the alloy, improves the plasticity of Hf_0.25_NbTa_0.25_TiZr alloy, and promotes crystal line defects to improve the strength of the alloy. LMD–DCT has been proven to be an effective method of synergistically improving the strength and plasticity of the alloy. Furthermore, DCT with a long immersion time can avoid a large degree of processing deformation and directly promote the formation of HCP precipitates near the grain boundary position. The presence of precipitates further improves the strength of the alloy.

The DCT method can effectively utilize the residual stress accumulated in the alloy during the forming process of laser additive manufacturing methods such as LMD. Compared with traditional rolling methods, the DCT method is more suitable for the modification of complex shape parts formed by LMD near-net forming. For HfNbTaTiZr alloy, DCT can also be targeted for phase control. It can be said that DCT is an excellent supporting scheme for additive manufacturing. Based on the DCT-induced phase transition found in this study, increasing the density of grain boundaries by means of selective laser melting to increase the density of precipitated phases will also be the direction of our further exploration.

## Data Availability

Date are contained within the article.
